# A genome-wide investigation of *Mycoplasma hominis* genes associated with gynecological infections or infertility

**DOI:** 10.3389/fmicb.2025.1561378

**Published:** 2025-04-30

**Authors:** Salim Chibani, Elhem Yacoub, Safa Boujemaa, Helmi Mardassi, Julien Guglielmini, Amaury Vaysse, Nadine Khadraoui, Béhija Mlik, Boutheina Ben Abdelmoumen Mardassi

**Affiliations:** ^1^Group of Mycoplasmas, Laboratory of Molecular Microbiology, Vaccinology, and Biotechnological Development, Pasteur Institute of Tunis, University of Tunis-El Manar, Tunis, Tunisia; ^2^Unit of Typing and Genetics of Mycobacteria, Laboratory of Molecular Microbiology, Vaccinology, and Biotechnology Development, Pasteur Institute of Tunis, University of Tunis-El Manar, Tunis, Tunisia; ^3^Institut Pasteur, Université Paris Cité, Bioinformatics and Biostatistics Hub, Paris, France

**Keywords:** *Mycoplasma hominis*, pathotype, infertility, gynecological infections, virulence, whole genome sequencing, genome-wide association studies

## Abstract

**Background and aim:**

*Mycoplasma hominis* is a human pathogenic bacterium that causes a wide range of genital infections and reproductive issues. Previously, based on an extended multilocus sequence typing scheme, we provided evidence for the segregation of *M. hominis* clinical strains into two distinct pathotypes: gynecological infections or infertility. Here, based on whole genome sequencing (WGS) data, we sought to provide a more refined picture of the phylogenetic relationship between these two *M. hominis* pathotypes, with the aim to delineate the underlying genetic determinants.

**Methods:**

We carried out WGS of 62 Tunisian *M. hominis* clinical strains collected over a 17-year period. The majority of these clinical strains are associated with infertility (*n* = 53) and the remaining nine isolates are from gynecological infections cases. An alignment-free distance-based procedure (Jolytree) was used to infer phylogenetic relationships among *M. hominis* isolates, while the phylogenetic method treeWAS was used to determine the statistical association between pathotypes of interest and genotypes at all loci.

**Results:**

The total pangenome of *M. hominis* strains was found to contain 1,590 genes including 966 core genes and 592 accessory genes, representing 60 and 37% of the total genome, respectively. Collectively, phylogenetic analyses based on WGS confirmed the distinction between the two *M. hominis* pathotypes. Strikingly, genome wide association analyses identified 4 virulence genes associated with gynecological infections, mainly involved in nucleotide salvage pathways and tolerance to oxidative stress, while five genes have been associated with infertility cases, two of which are implicated in biofilm formation.

**Conclusion:**

In sum, this study further established the categorization of *M. hominis* into two pathotypes, and led to the identification of the associated genetic loci, thus holding out promising prospects for a better understanding of the differential interaction of *M. hominis* with its host.

## Introduction

*Mycoplasma hominis* belongs to the genus of *Mycoplasma*, the class of *Mollicutes*, and the family of *Mycoplasmataceae* (Pettersson et al., 2000). It was the first mycoplasma isolated from humans in 1937 (Dienes and Edsall, 1937). Like all Mollicutes species, *M. hominis* lacks the cell wall which makes it naturally resistant to antibiotics targeting the cell wall such as the beta-lactams and glycopeptides (Chernova et al., 2016). The genome size of *M. hominis* reference strain PG21 (ATCC 23114) was estimated to be around 665 kb (Calcutt and Foecking, 2015), thus exceeding *Mycoplasma genitalium* genome (580 kb), the smallest genome of any organism that can be grown in pure culture (Fookes et al., 2017), by only 85 kb. *M. hominis* is subject to high variability, allowing it to adapt to the host environment (Boujemaa et al., 2018). This variability mainly occurs in surface proteins including the variable adherence-associated protein (Vaa) (Boesen et al., 2004, 2001), the P120 surface protein and the surface-located membrane protein (Lmp) family (Ladefoged et al., 1996; Ladefoged and Christiansen, 1998). *M. hominis* colonizes the mucosa of the urogenital tract of healthy persons as a commensal bacterium (Elias et al., 2005). However, its presence at high titers can cause a variety of diseases like urinary tract infections, bacterial vaginosis, pelvic inflammatory disease, cervicitis, and pyelonephritis (Kataoka et al., 2006; Küchle et al., 1997). Along with other genital mycoplasmas, it was demonstrated that *M. hominis* can be the origin of a wide array of infectious diseases, leading to infertility in women (Abdulrazzak and Bakr, 2000) and men (Huang et al., 2014, 2015). Indeed, it has previously been shown that *M. hominis* is present in 37.5% of infertile males and females (Jamalizadeh-Bahaabadi et al., 2014). *M. hominis* was also frequently isolated from infected fetal membranes and amniotic fluid (Allen-Daniels et al., 2015; Osei Poku, 2022). Microbial invasion of amniotic fluid is indeed the main contributor to poor pregnancy outcomes (Jain et al., 2022). Apart from genital infections, several reports have evoked the association of *M. hominis* with extragenital infections in different anatomical localizations like the central nervous system, the joints, and the heart, especially in immunocompromised patients (Yang et al., 2022; Luttrell et al., 1994).

In a previous work conducted in our laboratory, based on an extended multilocus typing (eMLST) scheme, we demonstrated that *M. hominis* can evolve into two distinct pathotypes (gynecological infections and infertility) by accumulating both mutations and recombination events (Boujemaa et al., 2018). However, there is as yet no study that attempted to establish an association between genetic loci and a specific pathotype, despite the publication of several *M. hominis* genomes from various countries (Calcutt and Foecking, 2015; Meygret et al., 2019; Allen-Daniels et al., 2015). Prokaryotic genome wide association tools have been shown to be reliable enough for identifying genetic loci related to phenotype (Read and Massey, 2014). This has prompted us to undertake this study aiming at determining the specific genetic factors behind the segregation of *M. hominis* strains into pathotypes and to gain more knowledge about the evolutionary dynamics of this species. Genomewide association tools would highlight the potential selective pressures acting on *M. hominis* clonal evolution and adaptation to cause a particular clinical manifestation.

## Materials and methods

### Clinical strains

This study involved 62 Tunisian *M. hominis* clinical strains collected over a period of 17 years (from 2000 to 2017) from gynecological consulting centers based in Tunis. Ten strains were isolated from patients having gynecological infections using vaginal swabs and 52 strains were isolated from both male and female patients showing infertility signs. More details about epidemiological characteristics of *M. hominis* strains are given in the Supplementary Table 1. The genomic sequence of the *M. hominis* reference strain PG21 was downloaded from NCBI database and used for annotation and genomic comparisons (Genbank accession number GCA_000085865.1). All strains were grown in SP4 broth and solid medium as described elsewhere (Tully et al., 1983). Then, they were cloned three times from single colonies resulting from plating of limiting dilutions (Boujemaa et al., 2018). To further confirm the *M. hominis* identity of the isolated mycoplasma colonies, PCR was performed as previously described (Ben Abdelmoumen Mardassi et al., 2007). Whole genomic DNA was extracted according to the phenol-chloroform protocol (Dale and Greenaway, 1985) from *M. hominis* culture obtained from a single colony of the third cloning step as described elsewhere (Ben Abdelmoumen and Roy, 1995). Subsequently, DNA samples were quantified using Qubit (high broad range buffer) then sent for sequencing at the Biomics platform, Institut Pasteur, Paris.

### Sequencing, assembly, and annotation

Whole genomes of *M. hominis* strains were sequenced using Illumina MiSeq with the 2× 150 bp sequencing configuration. FastQ and MultiQC softwares were used to verify the quality of the generated reads. A freely available in-house pipeline, fq2dna (https://gitlab.pasteur.fr/GIPhy/fq2dna), was employed to perform high quality *de novo* assembly. This pipeline includes several softwares, each one being used for a specific function. SPADES was used for genome assembly of short reads (Bankevich et al., 2012). Platon for the identification of contigs belonging to a chromosome or a plasmid (Schwengers et al., 2020), and Minimap2 for the alignment of query sequences against reference genome sequence (Li, 2018). Another in-house pipeline responsible for reads pre-preprocessing (fqCleanER: https://gitlab.pasteur.fr/GIPhy/fqCleanER) that includes the following softwares: fqtools (https://www.ncbi.nlm.nih.gov/pmc/articles/PMC4908325/) for reads deduplication, AlienTrimmer (https://pubmed.ncbi.nlm.nih.gov/23912058/) for reads trimming and clipping, FLASH (https://pubmed.ncbi.nlm.nih.gov/21903629/) for reads merging, and Musket (https://academic.oup.com/bioinformatics/article/29/3/308/257257) for sequencing error correction. Finally, genome annotation was performed using rapid prokaryotic genome annotation (Prokka) software (Seemann, 2014).

### Pangenome analysis

Panaroo (Tonkin-Hill et al., 2020) was applied to study pan, core, and accessory genomes of *M. hominis*. The pangenome designates the entire set of genes present in a particular bacterial set of genomes that can be divided into the core and the accessory genome. The core genome is composed of the conserved genes that are present among 95 to 100% of strains belonging to the same species. However, the accessory genome is highly variable and contains both shell genome representing the set of genes that are present in 15 to 95% of strains and cloud genome representing the set of genes that are present in 0 to 15% of strains (Brockhurst et al., 2019; Tonkin-Hill et al., 2020). Panaroo was also used to establish structural variations of genes (duplication, deletion, copy number) along sequence length of *M. hominis* from 1 to 3 Kb.

R studio package was used to generate pangenome matrix from gene presence/absence table of Panaroo (Tonkin-Hill et al., 2020). Classification of genes or clusters of orthologous genes (COG) was performed using the bacterial pangenomic pipeline BPGA that supports protein sequence files of Prokka as inputs (Chaudhari et al., 2016).

### Phylogenetic and phylodistribution analysis

The phylogenetic method treeWAS was used to determine the statistical association between pathotypes of interest and genotypes at all loci (Collins and Didelot, 2018). TreeWAS used 3 kinds of tests: terminal, simultaneous, and subsequent. The terminal test is a sample-wide test of association that seeks to identify broad patterns of correlations between genetic loci and the phenotype, without relying on inferences drawn from reconstructions of the ancestral states (Collins and Didelot, 2018). The simultaneous test allows for the identification of simultaneous substitutions in both the genetic locus and phenotype on the same branch of the phylogenetic tree. Simultaneous substitutions are an indicator of a deterministic relationship between genotype and phenotype. Score threshold is determined to separate genes with high substitution rates from others, which can estimate the degree of parallel change in the phenotype and genotype across branches of the tree (Collins and Didelot, 2018). The subsequent test measures the proportion of the tree in which the genotype and phenotype co-exist by drawing on inferences from the ancestral state reconstructions. The groups of genes that have a score higher than the threshold of 0.7 are considered significantly associated with one of the two pathotypes. The association may happen with the presence or the absence of the gene. Presence is indicated by a score of 1 and absence by a score of 0.

Pyseer (Lees et al., 2018) was used to filter genetic variants and to select only the highly significant loci with the pathotypes of interest. K-mers of variable length counted from draft assemblies are used as the input, and their association with a phenotype of interest is assessed by fitting a generalized linear model to each k-mer.

### Alignment and comparative genomics

In order to curate annotation outputs and to conduct comparative genomic analysis, BLAST (https://blast.ncbi.nlm.nih.gov/Blast.cgi) and ClustalW2 (Thompson et al., 1994) were used to generate nucleotide and protein multiple sequence alignments. Nucleotide BLAST (BLASTN) was used to align pathotype-associated genes detected in our 62 *M. hominis* sequences with all those available in NCBI database (https://www.ncbi.nlm.nih.gov/genbank/). Further comparative genomic analyses were conducted using Molligen Database (http://cbi.labri.fr/outils/molligen/), InterProScan database (http://www.ebi.ac.uk/interpro/), and VFDB (virulence factor database) (http://www.mgc.ac.cn/VFs/).

We used protein BLAST analysis for the proteins which were not assigned using Prokka software (listed below), to confirm their annotation.

- Vaa variable adherence-associated gene: Genbank number U56828.1.
- P60 (ORF1)-P80 (ORF2) complex: Genbank number Z29068.2.
- P120 surface protein: Genbank number U22019.1.
- P120′ surface protein: Genbank number MG879427.1.
- Lmp family of surface proteins: Genbank number VJ45_RS01770.

### Topology prediction of new proteins

Trans-membrane topography prediction was performed using the PSIPREPD Protein Sequence Analysis Workbench (http://bioinf.cs.ucl.ac.uk/psipred/). TMHMM (https://dtu.biolib.com/DeepTMHMM) was used to predict the localization of proteins of interest and to identify their surface, transmembrane, and inner membrane domains.

### Single nucleotide polymorphism (SNP) analysis

Assembled Fasta files were aligned to *M. hominis* PG21 reference genome using Minimap2 (Li, 2018). The generated Sam files were converted to Bam files using Samtools package (Ramirez-Gonzalez et al., 2012). Then, BCFtool was employed to call SNPs at all loci to generate final VCF file that was analyzed using BCFtoolstats (Danecek and McCarthy, 2017). Student's *t*-test was used to assess statistical significant difference (*p* = 0.0055) between the mean SNPs count values of strains linked to infertility and gynecological infections (*P* > 0.05).

## Results

### Genomic features of *M. hominis* strains as revealed by high throughput sequencing

FastQC showed a high-quality mapping with a Phred score higher than 36 indicating a high percentage of identified bases and <0.01% of over represented sequences on average, indicating a low percentage of adaptater contamination and a high coverage sequencing (20×). Analysis revealed a low percentage of duplicated sequences (between 2.5 and 2.8%) and high proportion of properly mapped reads spanning between 79.23% (MH61 strain) and 99.88% (MH62 strain), with a medium value of 88.05%. Genome assembly using Spades resulted in various numbers of contigs. The best assembled genome was observed in MH6 strain with only three contigs, whereas the most fragmented genome was found in the strain MH61 with 41 contigs. The WGS results revealed significant similarity in genomic characteristics between *M. hominis* clinical strains and the reference strain PG21, with a genome length ranging between 656 421 bp and 752 582 bp compared to the 665 445 bp length of the reference strain. GC content percentages calculated in all clinical strains slightly vary between 26.84 and 27.21% and were very close to the overall GC content in *M. hominis* reference strain PG21 (27.12%). Mean number of CDSs was 1,173, which is slightly higher than that observed for the reference strain (CDSs = 1,113). Like strain PG21, genes encoding for ribosomal RNA (rRNA) are organized in one operon having multiple copies (1 to 4). We found 31 to 34 gene copies encoding for transfer RNA (tRNA) and only one transfer-messenger RNA (tmRNA) gene. All these results are summarized in Supplementary Table 2.

### COG enrichment analysis

**Figure 1:**
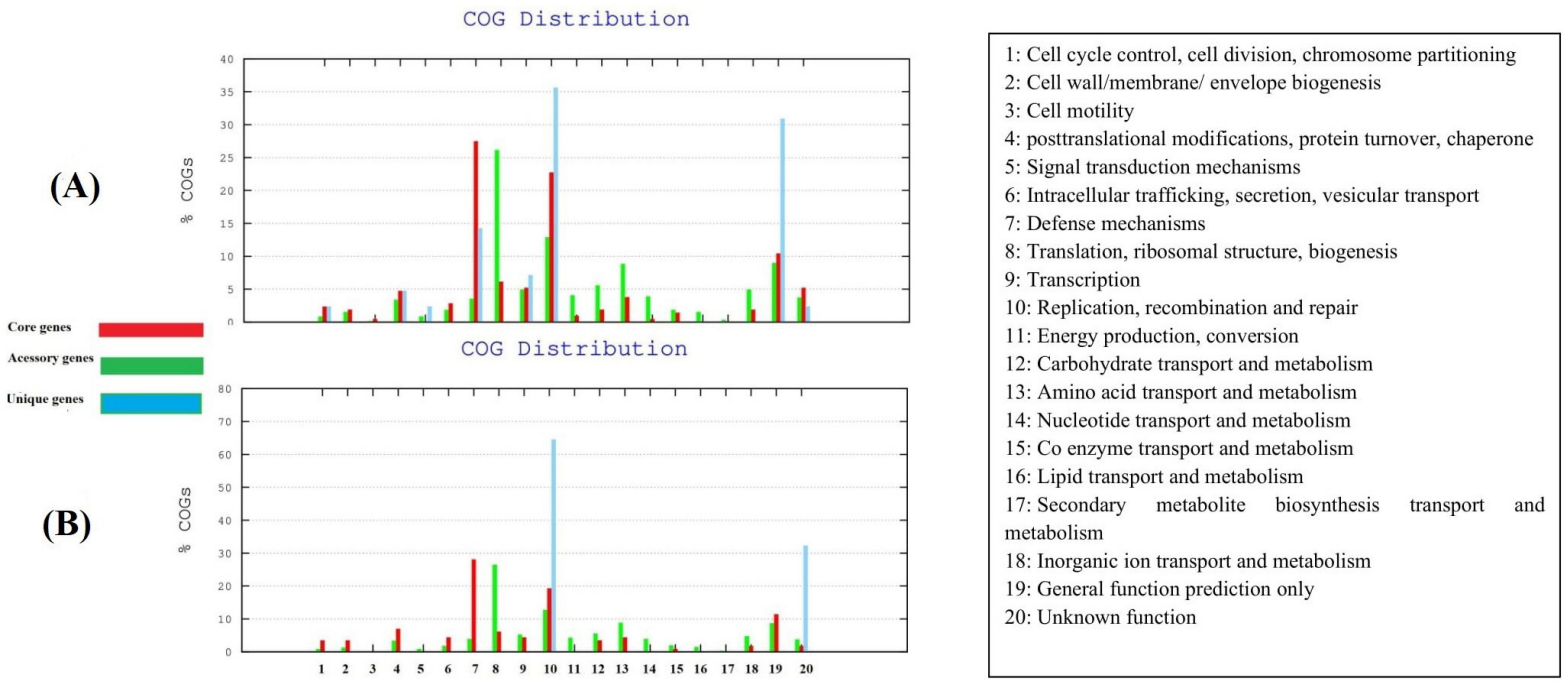
COG classification of genes. **(A)** Clusters of orthologous genes (COGs) distribution among the 52 *M. hominis* strains associated with infertility. **(B)** COGs distribution among the 10 *M. hominis* strains associated with genital infections. Bars in green, red, and blue, represent the core genome, the accessory genes (shell genes), and unique genes (cloud genes), respectively.

Among the 51 *M. hominis* isolates linked to infertility, we found that 25% of the core genome was composed of genes encoding for translation ribosomal structure and biogenesis, 15% involved in DNA repair and replication, and 10% belonged to energy metabolism genes. The major categories included in the accessory genome, are genes involved in defense mechanisms and virulence (28%), replication and transcription (5%). Unique genes include mainly genes involved in replication, recombination, and repair (35%) (Figure 1A). As for the nine *M. hominis* strains associated with gynecological infections, the quarter of the core genome is involved in ribosomal structure and biogenesis, with 10% encoding for ion transport and metabolism. Almost one-third (29%) of the accessory genome was found to encode for virulence genes, and 60% of the shell genome comprised unique genes involved in DNA repair and recombination. Cell motility unique genes were more prevalent among this pathotype (Figure 1B).

### Pangenome distribution

According to Panaroo results, the pangenome of *M. hominis* isolates comprised a total of 1590 genes. The core, soft, shell, and cloud genomes included 966, 32, 359, and 233 genes, respectively (Supplementary Table 3). Together, shell and cloud genomes comprised 592 genes, accounting for almost 37% of the total pan genome, a finding that was confirmed by pangenome distribution matrix (Supplementary Figure 1).

### Single nucleotide polymorphism analysis

**Figure 2:**
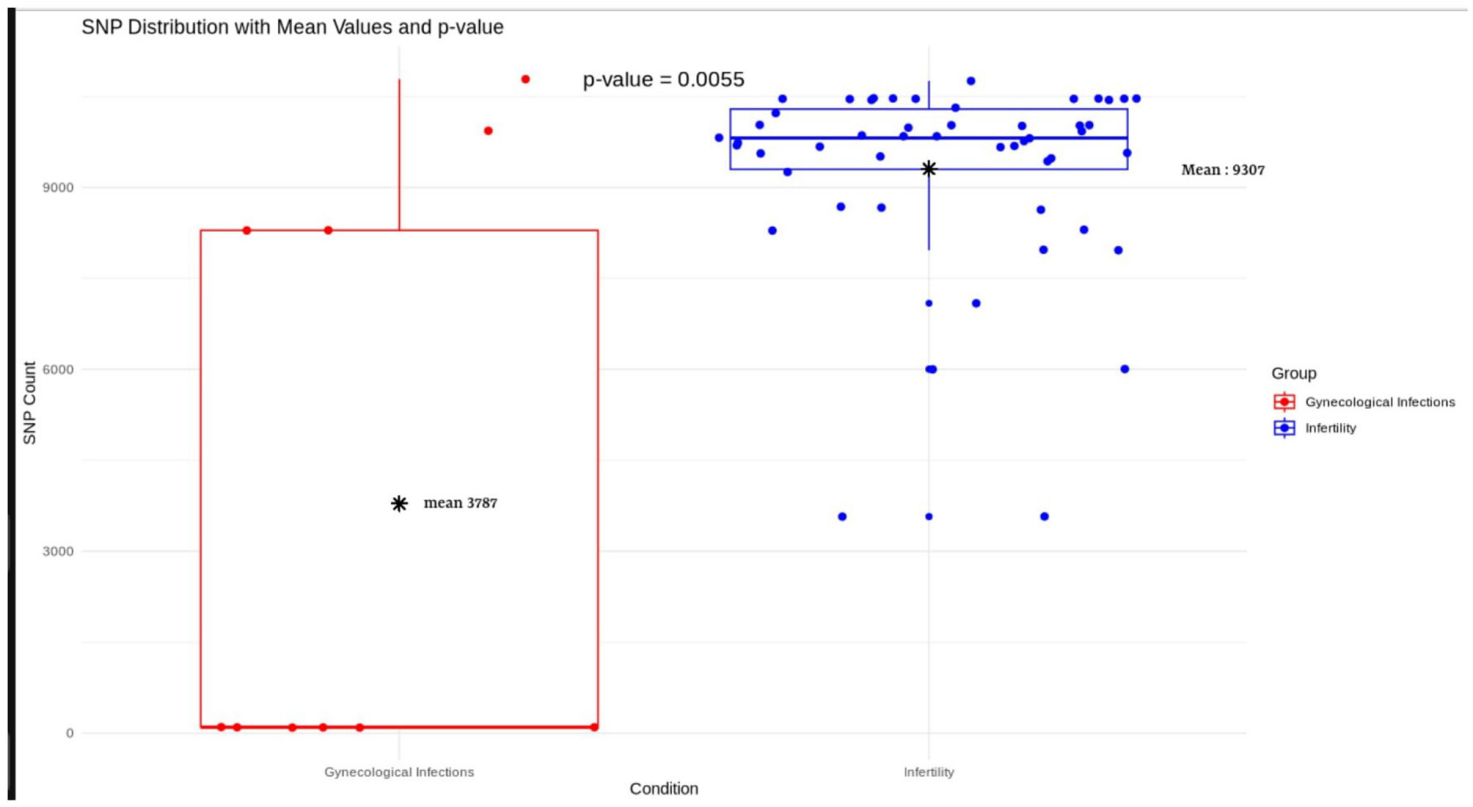
Single nucleotide polymorphism (SNP) distribution among *M. hominis* strains associated with gynecological infections (red boxplot) and infertility (blue boxplot). Student's *t*-test was used to assess for statistical significant difference (*P* < 0.05) between the mean SNPs count values of strains linked to infertility and gynecological infections. ^*^Represents the mean SNP count of each boxplot.

Comparative analysis of genomic SNPs counts among *M. hominis* strains associated with gynecological infections showed that while some strains (*N* = 5) displayed low number of SNPs, ranging between 91 and 100, the remaining four strains showed a high number of SNPs, varying between 8,291 and 10,786. By contrast, *M. hominis* strains linked to infertility tended to accumulate a greater number of SNPs, ranging from 2,880 to 8,867. Statistical analysis using Student's *t*-test indicated a significant difference (*p* = 0.0055) between the mean SNP values of infertility-associated strains (9,307) and those associated with gynecological infections (4,289) (Figure 2). Additionally, transition substitution mutations were found to occur at a higher frequency compared to insertions and deletions in both pathotypes, a pattern consistent with the mutational feature observed in many bacterial species (Supplementary Table 3).

### Characterization of the two *M. hominis* pathotypes

**Figure 3:**
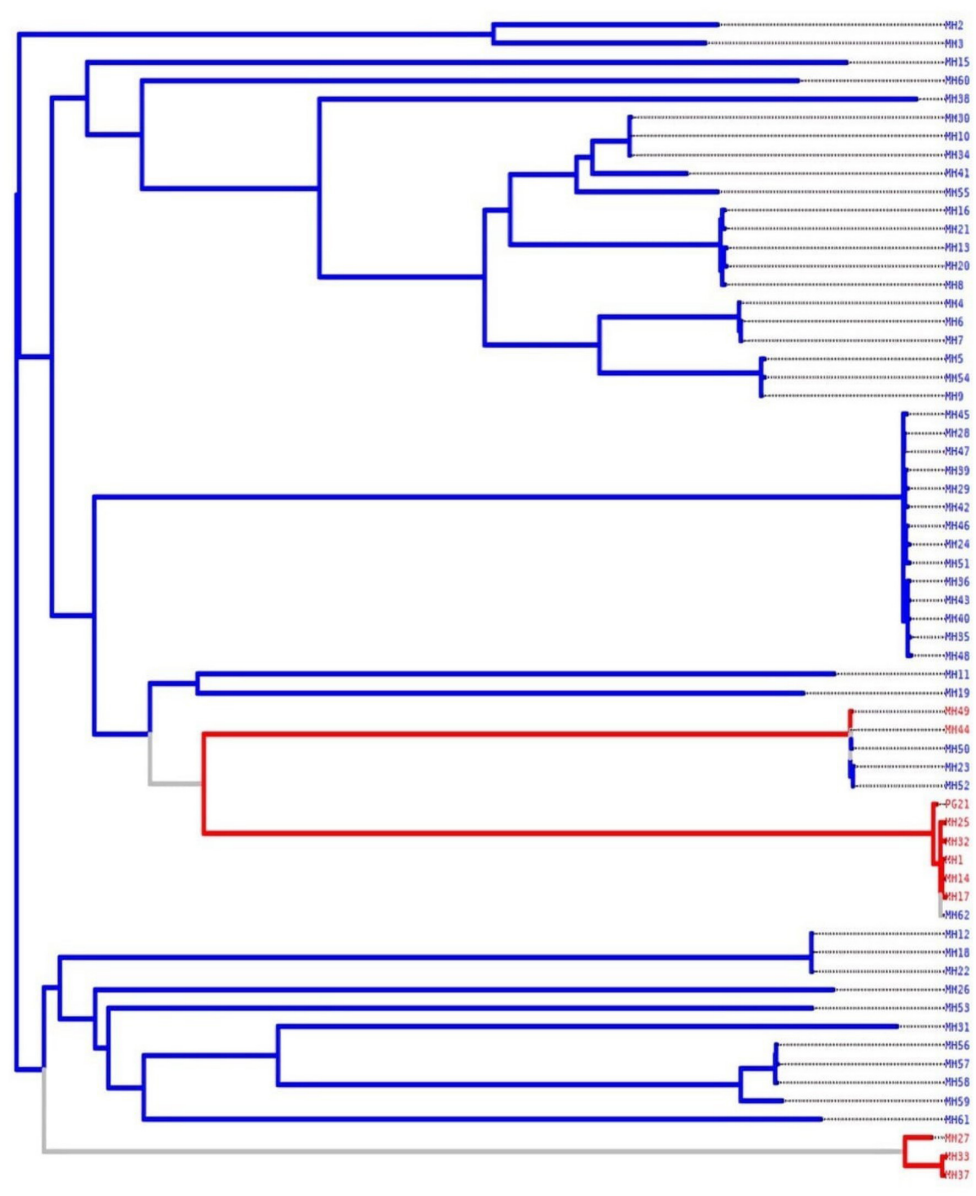
TreeWAS-based phylogenetic tree illustrating the relatedness between the 62 Tunisian *M. hominis* clinical strains based on pathotype association. Strains in red are associated with gynecological infections pathotype and strains in blue are associated with infertility pathotype.

TreeWAS results demonstrated a clear categorization of *M. hominis* clinical strains into two distinct lineages, each one referring to a specific pathotype. Indeed, the nine strains recovered from patients presenting with gynecological infections were assigned the same pathotype. Likewise, the 52 strains linked to infertility cases were associated with the same pathotype (Figure 3).

Phylogenic relatedness of *M. hominis* strains based on long sequence variations (1kb, 3kb) showed that the reference strain PG21 and the clinical strains MH1, MH17, MH32, MH14, and MH25 were associated with gynecological infections (data not shown), a finding confirmed by the phylogenetic tree (Figure 3).

### Identifiying loci associated with infertility or gynecological infections pathotype

**Figure 4:**
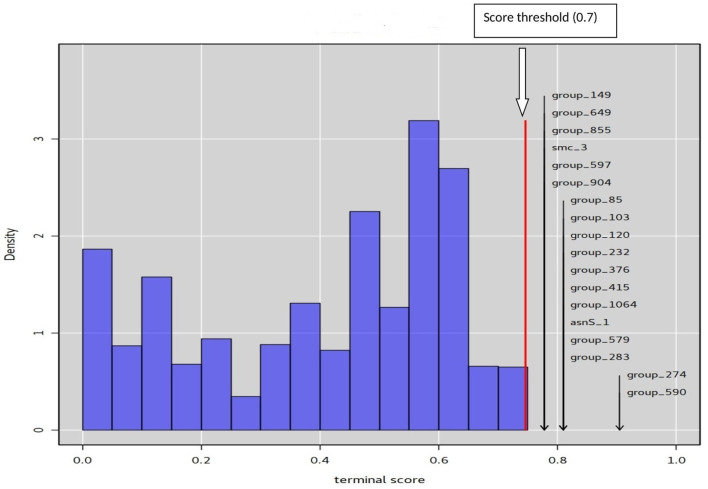
Null distributions of simulated association terminal scores. The red vertical line marks the significance threshold (0.7), above which real associated genes are indicated.

Based on a gene presence or absence matrices, TreeWAS terminal test analysis identified 18 gene loci significantly associated (score threshold > 0.7) with gynecological infections or infertility pathotypes (Figure 4, Supplementary Figure 2). Interestingly, among these, 4 gene sequences also proved significantly associated with the gynecological infections pathotype in the TreeWAS subsequent test (Table 1). These genes encode for an asparagine-tRNA ligase (asnS), a restriction endonuclease subunit S containing DNA methylase domain and Eco47II endonuclease (Group 1,064), an Eco47II family restriction endonuclease that is involved in degradation of host DNA (Group 590), and XRE, a transcription regulator, which is involved in tolerance to oxidative stress (Group 274) (Virulence factor database).^1^

**Table 1:** Gene table of significant loci using TreeWas subsequent test.

**Gene sequence associated with the pathotype of interest**	**Correlations statistics**		**Infertility pathotype**		**Gynecological infections pathotype**		**Reference strain PG21**
**ID of the locus in Panaroo outputs**	***P*-value**	**S Score**	**Gene presence**	**Gene absence**	**Gene presence**	**Gene absence**	**Corresponding locus (position in bp) if present**
Group_283	0	−0.8095238	51	1	5	6	-
Group_904	0	−0.7777778	50	2	5	6	-
Group_597	0	−0.7777778	50	2	5	6	-
Group_579	0	−0.8095238	46	6	0	11	FP236530.1 (557,253..557,436)
asnS_1	0	0.8095238	6	46	11	0	-
Group_1064	0	0.8095238	3	49	8	3	-
Group_590	0	0.9047619	1	51	9	2	-
Group_274	0	0.9047619	1	51	9	2	FP236530.1 (46,571..46,771)
smc_3	0	0.7777778	2	50	6	5	FP236530.1 (365,509..368,976)
Group_855	0	0.7777778	2	50	6	5	FP236530.1 (13,577..13,768)
Group_649	0	0.7777778	2	50	6	5	FP236530.1 (648,886..649,224)
Group_149	0	0.7777778	2	50	6	5	FP236530.1 (648,134..648,478)
Group_415	0	−0.8095238	1	51	6	5	FP236530.1 (601,335..601,430)
Group_376	0	0.8095238	1	51	6	5	FP236530.1 (13,577..13,768)
Group_232	0	0.8095238	1	51	6	5	FP236530.1 (48,987..49,220)
Group_120	0	0.8095238	1	51	6	5	FP236530.1 (600,894..601,286)
Group_103	0	0.8095238	1	51	6	5	FP236530.1 (272,880..273,305)
Group_85	0	0.8095238	1	51	6	5	-

*P* value < 0.05 indicates significant association. S score: the score value for significant association loci. S > 0.7 indicates association of the gene locus with one of the two pathotypes.

Regarding the infertility-associated pathotype, terminal test analysis detected five genes encoding for two putative lipoproteins (Group 283 and Group 597), a hypothetical protein (Group 904), a glycosyl transferase (Group 579), and a pyruvate kinase (pyk). The two latter proteins are involved in biofilm formation (Molligen database)^2^ (Figure 4; Supplementary Figure 2).

Next, TreeWas simultaneous test was used to identify parallel change in the phenotype and genotype across branches of the tree that could be associated with the infertility or gynecological infections pathotype. Significant signals of simultaneous substitution in phenotype and genotype was observed for gene segments deoB (phosphopentomutase), Group 359 (metalloenzyme), and pyk (the gene encoding for a pyruvate kinase required for biofilm formation), along with the infertility pathotype (Table 2).

**Table 2:** Gene table of significant loci using TreeWas simultaneous test.

**Genomic sequences undergoing simultaneous substitutions**	***P* value**	**S Score**	**Infertility**		**Gynecological infections**	
			**Gene presence**	**Gene absence**	**Gene presence**	**Gene absence**
Group_537-group_274-group_590	0	0.8730159	1	51	8	3
serS_3-pyk-group_453	0	0.8412698	3	49	9	2
deoB_1-group_359-pyk	0	0.8412698	49	3	2	9
deoB_1-group_359-group_453	0	0.8412698	3	49	9	2
pepC-group_537-group_274	0	0.8730159	1	51	8	3

*P* value < 0.05 indicates significant association. S score: the score values for significant association (Score > 0.7), indicating a simultaneous change (presence or absence) in gene locus and in one of the two pathotypes.

Likewise, four simultaneous substitutions in gynecological infections pathotype and various combinations of gene segments were significantly associated. These involved Group_537 (Cysteine peptidase)-group_274 (transcription regulator XRE)-group_590 (Eco47II family restriction endonuclease) (S score = 0.873), serS_3-pyk-group_453 (S score = 0.841), deoB_1-group_359 (metalloenzyme)-group_453 (S score = 0.841), and pepC-group_537-group_274 (oxidative stress) (S score = 0.873) (Table 2).

## Discussion

*M. hominis* has been shown to contribute to many pathologies in humans (Ahmed et al., 2021). This mycoplasma species was found to be responsible for distinct pathological conditions, notably infertility and gynecological infections (Boujemaa et al., 2018; Cheng et al., 2023). The existence of *M. hominis* pathotypes associated with the two latter conditions has been evoked earlier by our group, based on an extended multilocus sequence typing scheme (Boujemaa et al., 2018). Here, we extended our investigation to the whole genome in order to be able to carry out reliable phylogenetic and association studies.

Despite the relatively small number of isolates included in this study (62 stains), GWAS analysis unambiguously distinguished *M. hominis* clinical strains into two distinct pathotypes (infertility and gynecological infections), thus confirming our previous, eMLST-based results, which included a combination of housekeeping (gyrB, tuf, ftsY, uvrA, and gap) and virulence (p120′, vaa, lmp1, lmp3 and p60) genes (Boujemaa et al., 2018). Another molecular typing methods using MLVA (multilocus variable tandem repeat) has also confirmed this trait in *M. hominis* French strains (Férandon et al., 2013). Here, we confirm this finding on a genome-wide scale.

We found that the two pathotypes differ significantly by the number of accumulated SNPs, with *M. hominis* strains involved in infertility displaying ~2 times more mutations than their counterpart linked to gynecological infections. This might be due to the chronicity of *M. hominis* infection in infertitlity cases. Indeed, it seems likely that during chronic infections, pathogens have sufficient time to generate multiple mutations. In Pseudomonas aeruginosa, for instance, higher mutation rates of the pathogen have been attributed, among others, to within-host chronicity that is characterizated by strong compartmentalization and low migration rates between hosts, which limit competition between strains, and thus preserve genetic variation (Denamur and Matic, 2006). Pathogen evolution during chronic infections has been particularly addressed in SARS-CoV-2 natural infection. It was shown that chronic infections lead to an increased rate of mutation accumulation compared to populations with only acute infections (Rodríguez-Horta et al., 2024). However, it remains to be seen to which extent these observations, which emanate from unrelated species, could apply to *M. hominis*.

Bacterial genome-wide association studies have been proposed as a useful approach to reveal the genetics of microbial phenotypes, particularly virulence (Yang et al., 2018). It is well known that bacteria reproduce clonally with many mutations and recombination events taking place, thus providing the raw material for selective pressure to form new pathotype associated variants (Dixit et al., 2017). This concept is considered as the cornerstone of the evolutionary biology considering the role of virulence factor combinations in a particular pathological context (Saber and Shapiro, 2020). The simultaneous presence of virulence factors can exert synergistic effect and enhance bacterial virulence through adherence and host damaging effects of endotoxins (Sharma et al., 2017) as shown in *Helicobacter pylori* (Rad et al., 2003). For mycoplasmas as well, it has been demonstrated that virulence can be enhanced through simultaneous presence of proteases, nucleases, and oxidative stress tolerance genes (Yiwen et al., 2021). In our study, the simultaneous detection of a set of 4 virulence factors (Asparagine tRNA ligase, restriction endonuclease subunit S, Eco47II family restriction endonuclease, and transcription regulator XRE) was found to be indicative of the gynecological infections-associated pathotype. These genes are widely distributed in Gram-positive bacteria (Loenen et al., 2014). XRE transcription regulator has been shown to play a major role in the restriction modification system of the avian pathogenic mycoplasma species *Mycoplasma gallisepticum* (Semashko et al., 2022) (Molligen database; see text footnote ^2^) and its role in virulence and oxidative stress tolerance has been demonstrated in other bacteria such as *Streptococcus suis* (Hu et al., 2019). Eco47II family restriction endonuclease has been annotated in Mycoplasma felis and *Mycoplasma mycoides* as part of restriction modification systems that protect the genome against incoming mobile elements (Klose et al., 2024). Aminoacyl-tRNA synthetases represented by asparagines tRNA ligase seem to be essential for proper translation and adaptation to various stress-related conditions (Rubio Gomez and Ibba, 2020). Simultaneous substitutions can also be a potent driver of change in gene function leading to evolution to a novel pathotype. For instance, substitutions in cysteine peptidase (Goup 537) is here correlated with the gynecological infections-associated pathotype. In *Streptococcus thermophiles*, the homologous cysteine aminopeptidase (Chapot-Chartier et al., 1994) has been shown to have proteolytic activity that allows bacteria to degrade peptides (Yang et al., 2023). Cysteine proteases are prevalent among *Lactobacillus* species and allow for the degradation of milk peptides (Rodríguez-Serrano et al., 2018). The presence of such proteases in *M. hominis* can be explained by the deficiency in amino-acid synthesis pathway (Yu et al., 2009).

As for the compensation of deficiencies in nucleic acid precursor synthesis, nucleotide salvage pathways have been frequently described in mycoplasmas allowing them to rely on exogenous supply from the host in order to synthetize purines and pyrimidines (Wang et al., 2001; Cacciotto and Alberti, 2022). Such enzymes with endonuclease activity have been detected in human mycoplasmas such as *Mycoplasma pneumoniae* and *Mycoplasma arginini* (Wang et al., 2001; Gioia et al., 2023). The increasing scientific knowledge about endonucleases has already provided insights into their implication in many pathological processes in the host cells such as DNA damage, apoptosis, cytotoxicity, and biofilm formation (Yiwen et al., 2021; Williams, 2003; Sharma et al., 2017). Such association with various pathological processes can indirectly contribute to gynecological infections. Indeed, nucleases have been shown to play a role in adaptative response (Gonzalez and Hernandez, 2022) and degradation of neutrophil extracellular trap (Steichen et al., 2011). NucA (a divalent metal ion-dependent nucleases) have been shown to be implicated in the logarithmic growth of *Serratia marcescens*, which is critical during infection.

Regarding the set of the five genes associated with the infertility pathotype, two were found to encode for putative lipoproteins named Group 283 and Group 597, and they seem to be specific to *M. hominis* species as they were not detected in other mycoplasma species. Transmembrane topology prediction revealed that both putative lipoproteins possess an extracellular domain suggesting that they could have a role in adherence to host tissues. In *Mycoplasma genitalium*, adhesion proteins, MgpB and MgpC, are both required for formation of an attachement organelle (Burgos et al., 2006; Collier et al., 1990; Iverson-Cabral et al., 2015). The association of these putative lipoproteins with the infertility pathotype goes in line with previous studies showing the involvement of several other bacterial surface lipoproteins in infertitily. Indeed, these proteins can mimic human proteins and, hence, elicit an autoimmune response leading to infertility (Thaper and Prabha, 2018). In addition, in silico search for conserved motifs in Group 283 (putative lipoprotein) identified seripauperin and TIP1 family conserved domains (Interproscan database).^3^ In *Saccharomyces cerevisiae*, seripauperin proteins have previously been shown to play a role in stress response, especially under low temperature and anaerobiosis and, thus, to have a possible role in virulence (Luo and Van Vuuren, 2008). Moreover, the association of another gene, Group 579, with infertility seems not to be a fortuitous finding. Indeed, this gene encodes a glycosyltransferase, which is considered an important virulence factor in urogenital mycoplasmas. In mycoplasmas colonizing the upper respiratory tract such as *Mycoplasma pneumoniae*, glycosyltransferases encoded by Mpn483 are involved in various virulence processes using other fuel substrates such as UDP glucose and ceramides to form galactrocerbroside, which is involved in autoimmune reactions (Gaspari et al., 2021). In *Mycoplasma genitalium*, glycosyltransferase is essential for the synthesis of membrane glycolipids (Romero-García et al., 2019). It has been demonstrated that this enzyme plays a role in the formation of polysaccharides (Schmid et al., 2016; Zabotina et al., 2021), which contribute to escaping the immune system detection by resisting phagocytosis (Marques et al., 2016; Jank et al., 2015). Many components including exopolysaccharides (Daubenspeck et al., 2020), DNA, and polypeptides form biofilm matrix allowing mycoplasmas to escape from phagocytosis and, hence, immune surveillance (Yiwen et al., 2021), ensuring the chronicity of infections, which, in turn, may lead to infertility. Furthermore, the role of biofilm in promoting chronic infections is established and its formation can be reinforced by the upregulation of the expression of energy metabolism enzymes such as pyruvate dehydrogenase complex and the elongation factor (EF-Tu), as well as extracellular matrix-binding adhesions (Yiwen et al., 2021). In *M. hominis*, pyruvate kinase expression is increased in strains that have a slow growth during biofilm formation and under condition of thymidine fuel switching (Evsyutina et al., 2022). In the case of our *M. hominis* strains, we have noticed the absence of pyruvate dehydrogenase and the presence of the protein EF-Tu and pyruvate kinase. The dual role of these proteins in metabolism and biofilm formation have been hypothesized by other studies that highlighted the high expression level of these genes during biofilm formation like, for example, in Staphylococcus aureus bacterial infections (Vasu et al., 2015). Many possible mechanisms that may establish a link between biofilm formation and infertility in both females and males have been suggested, including the acquisition of some virulence genes like sialidase gene or the disruption of genital flora homeostasis (Swidsinski et al., 2013). In fact, biofilm formation has a strong influence on female vaginal microbiome homeostasis via complex interactions, which may lead to vaginal dysbiosis and most probably to infertility (Swidsinski et al., 2013; Machado et al., 2022; Moreno and Simón, 2021). Under certain conditions related to environmental stress and nutrients starvation, *M. hominis* is able to use thymidine as a fuel source (Semashko et al., 2020) through the expression of three genes: thymidine phosphorylase (deoA), phosphopentomutase (deoB), and deoxyribose-phosphate aldolase (deoC). This less advantageous pathway ensures slow growth and may also promote antibiotic resistance (Fisunov et al., 2022). This phenotypic switch from hydrolyzing arginine to thymidine may be associated with the survival of *M. hominis* inside the host as it is intriguingly unregulated during biofilm formation (Evsyutina et al., 2022). Several studies pointed out that hydrolyzing thymidine in the host by certain viruses like herpes simplex virus (HSV) contribute to the accumulation of thymidine kinase, which leads to male infertility via spermatogenesis disruption (Cai et al., 2009). In this study, we found that all thymidine hydrolyzing genes were conserved with the presence of simultaneous substitution in *deoB* gene, which is associated with *M. hominis* infertility pathotype. The implication of such event on gene expression needs further investigations.

In summary, analysis of the genome sequence of 62 *M. hominis* clinical strains, recovered from patients presenting with infertility disorders or gynecological infections, lends further support to the existence of a specific pathotype associated with each of these pathological conditions. The set of associated genes and/or parallel change in genotypes and phenotypes, as disclosed by the genome-wide association study, reflects the possible underlying mechanisms leading to the observed phenotypes.

## Data Availability

The datasets presented in this study can be found in online repositories. The names of the repository/repositories and accession number(s) can be found here: NCBI (https://www.ncbi.nlm.nih.gov/), accession number PRJNA1242796.

## Footnotes

^1^Virulence factor database: Available at: http://www.mgc.ac.cn/VFs/main.html.

^2^Molligen database: Available at: https://services.cbib.u-bordeaux.fr/molligen4/#!/.

^3^Interproscan Database: Available at: https://www.ebi.ac.uk/interpro/search/sequence/.
