# Supplementary material for: A genome-wide investigation of Mycoplasma hominis genes associated with gynecological infections or infertility
Source: Front Microbiol. 2025 Apr 30;16:1561378. doi: 10.3389/fmicb.2025.1561378 (PMC12075135; doi:10.3389/fmicb.2025.1561378)
Supplement: Supplementary file 1 [file Data_Sheet_1.docx]

***Supplementary Material***

**Supplementary Tables and Figures**

1. **Supplementary Tables**

**Supplementary table 1:** Origin of *Mycoplasma hominis* strains and patients clinical data

| Strain name | Year of isolation | Nature of specimens | Gender | Clinical manifestation |  |
| --- | --- | --- | --- | --- | --- |
| PG21 | 1953 | Rectal swab | Unknown | Healthy individual (https://www.atcc.org/products/23114) |  |
| MH1 | 2000 | Vaginal swab | Female | Gynecological infections |  |
| MH2 | 2006 | Semen | Male | Infertility |  |
| MH3 | 2010 | Vaginal swab | Female | Infertility |  |
| MH4 | 2005 | Vaginal swab | Female | Infertility |  |
| MH5 | 2003 | Vaginal swab | Female | Infertility |  |
| MH6 | 2002 | Vaginal swab | Female | Infertility |  |
| MH7 | 2001 | Vaginal swab | Female | Infertility |  |
| MH8 | 2000 | Semen | Male | Infertility |  |
| MH9 | 2010 | Vaginal swab | Female | Infertility |  |
| MH10 | 2008 | Semen | Male | Infertility |  |
| MH11 | 2007 | Semen | Male | Infertility |  |
| MH12 | 2005 | Semen | Male | Infertility |  |
| MH13 | 2005 | Vaginal swab | Female | Infertility |  |
| MH14 | 2003 | Semen | Male | Gynecological infections |  |
| MH15 | 2012 | Vaginal swab | Female | Infertility |  |
| MH16 | 2005 | Vaginal swab | Female | Infertility |  |
| MH17 | 2010 | Vaginal swab | Female | Gynecological infections |  |
| MH18 | 2006 | Semen | Male | Infertility |  |
| MH19 | 2011 | Semen | Male | Infertility |  |
| MH20 | 2007 | Vaginal swab | Female | Infertility |  |
| MH21 | 2014 | Semen | Male | Infertility |  |
| MH22 | 2010 | Vaginal swab | Female | Infertility |  |
| MH23 | 2012 | Semen | Male | Infertility |  |
| MH24 | 2004 | Vaginal swab | Female | Infertility |  |
| MH25 | 2015 | Vaginal swab | Female | Gynecological infections |  |
| MH26 | 2014 | Vaginal swab | Female | Infertility |  |
| MH27 | 2009 | Vaginal swab | Female | Gynecological infections |  |
| MH28 | 2006 | Vaginal swab | Female | Infertility |  |
| MH29 | 2010 | Vaginal swab | Female | Infertility |  |
| MH30 | 2008 | Vaginal swab | Female | Infertility |  |
| MH31 | 2015 | Vaginal swab | Female | Infertility |  |
| MH32 | 2010 | Vaginal swab | Female | Gynecological infections |  |
| MH33 | 2007 | Vaginal swab | Female | Gynecological infections |  |
| MH34 | 2003 | Vaginal swab | Female | Infertility |  |
| MH35 | 2008 | Vaginal swab | Female | Infertility |  |
| MH36 | 2012 | Vaginal swab | Female | Infertility |  |
| MH37 | 2011 | Vaginal swab | Female | Gynecological infections |  |
| MH38 | 2005 | Vaginal swab | Female | Infertility |  |
| MH39 | 2004 | Semen | Male | Infertility |  |
| MH40 | 2013 | Vaginal swab | Female | Infertility |  |
| MH41 | 2012 | Vaginal swab | Female | Infertility |  |
| MH42 | 2005 | Semen | Male | Infertility |  |
| MH43 | 2007 | Vaginal swab | Female | Infertility |  |
| MH44 | 2011 | Semen | Male | I |  |
| MH45 | 2016 | Vaginal swab | Female | Infertility |  |
| MH46 | 2013 | Vaginal swab | Female | Infertility |  |
| MH47 | 2011 | Vaginal swab | Female | Infertility |  |
| MH48 | 2009 | Vaginal swab | Female | Infertility |  |
| MH49 | 2011 | Vaginal swab | Female | Gynecological infections |  |
| MH50 | 2011 | Semen | Male | Infertility |  |
| MH51 | 2015 | Vaginal swab | Female | Infertility |  |
| MH52 | 2011 | Vaginal swab | Female | Infertility |  |
| MH53 | 2010 | Vaginal swab | Female | Infertility |  |
| MH54 | 2009 | Vaginal swab | Female | Infertility |  |
| MH55 | 2016 | Semen | Male | Infertility |  |
| MH56 | 2017 | Semen | Male | Infertility |  |
| MH57 | 2017 | Vaginal swab | Female | Infertility |  |
| MH58 | 2017 | Semen | Male | Infertility |  |
| MH59 | 2017 | Vaginal swab | Female | Infertility |  |
| MH60, | 2017 | Vaginal swab | Female | Infertility |  |
| MH61 | 2017 | Vaginal swab | Female | Infertility |  |
| MH62 | 2017 | Vaginal swab | Female | Infertility |  |

**Supplementary table 2:** Assembly statistics of *Mycoplasma hominis* strains

| Number of tmRNA | Number of tRNA | Number of rRNA | GC content (%) | Number of genes | Number of CDSs | Qualityof Mapping (%) | Mapped reads (%) | Number of contigs | Sequence length (bp) | Strain |  |
| --- | --- | --- | --- | --- | --- | --- | --- | --- | --- | --- | --- |
| 1 | 33 | 4 | 27.12 | 1157 | 1118 | 40.97 | 99.78 | 10 | 658894 | MH1 |  |
| 1 | 33 | 2 | 26.85 | 1225 | 1188 | 36.46 | 85.77 | 5 | 698508 | MH2 |  |
| 1 | 31 | 4 | 26.91 | 1205 | 1168 | 36.53 | 88.12 | 9 | 683343 | MH3 |  |
| 1 | 33 | 2 | 26.94 | 1150 | 1112 | 36.84 | 93.11 | 5 | 656728 | MH4 |  |
| 1 | 33 | 2 | 26.87 | 1276 | 1238 | 36.77 | 82.93 | 11 | 719478 | MH5 |  |
| 1 | 33 | 2 | 26.94 | 1147 | 1109 | 36.83 | 93.02 | 3 | 656421 | MH6 |  |
| 1 | 33 | 2 | 26.94 | 1147 | 1110 | 36.83 | 92.96 | 5 | 655930 | MH7 |  |
| 1 | 34 | 2 | 26.97 | 1198 | 1161 | 36.68 | 89.32 | 20 | 682661 | MH8 |  |
| 1 | 33 | 2 | 26.86 | 1275 | 1241 | 36.78 | 81.07 | 16 | 719446 | MH9 |  |
| 1 | 34 | 2 | 26.94 | 1187 | 1152 | 36.79 | 91.11 | 5 | 670448 | MH10 |  |
| 4 | 33 | 3 | 26.84 | 1174 | 1134 | 36.89 | 94.07 | 11 | 659821 | MH11 |  |
| 1 | 33 | 3 | 26.87 | 1247 | 1212 | 36.83 | 82.41 | 10 | 711137 | MH12 |  |
| 1 | 34 | 2 | 26.97 | 1203 | 1166 | 36.67 | 89.42 | 14 | 685811 | MH13 |  |
| 1 | 33 | 3 | 26.98 | 1157 | 1120 | 40.82 | 99.84 | 10 | 659750 | MH14 |  |
| 1 | 34 | 3 | 26.87 | 1262 | 1229 | 36.56 | 84.42 | 12 | 714311 | MH15 |  |
| 1 | 34 | 2 | 26.97 | 1192 | 1155 | 36.67 | 89.53 | 18 | 680685 | MH16 |  |
| 1 | 33 | 4 | 26.98 | 1160 | 1122 | 40.99 | 99.83 | 13 | 660930 | MH17 |  |
| 1 | 33 | 3 | 26.87 | 1245 | 1212 | 36.88 | 83.08 | 13 | 711793 | MH18 |  |
| 1 | 33 | 2 | 27.18 | 1186 | 1151 | 37.24 | 89.23 | 6 | 677428 | MH19 |  |
| 1 | 34 | 2 | 26.97 | 1198 | 1162 | 36.68 | 89.48 | 16 | 684257 | MH20 |  |
| 1 | 34 | 2 | 26.97 | 1191 | 1152 | 36.65 | 89.22 | 22 | 681104 | MH21 | |
| 1 | 33 | 3 | 26.87 | 1250 | 1214 | 36.9 | 82.6 | 12 | 711567 | MH22 | |
| 7 | 32 | 2 | 27.16 | 1173 | 1137 | 37.29 | 89.19 | 5 | 680880 | MH23 | |
| 1 | 33 | 3 | 27.11 | 1216 | 1179 | 36.92 | 86.86 | 6 | 714119 | MH24 | |
| 1 | 33 | 3 | 26.98 | 1160 | 1120 | 40.96 | 99.67 | 13 | 659996 | MH25 | |
| 1 | 33 | 3 | 26.90 | 1280 | 1243 | 36.36 | 84.67 | 10 | 731821 | MH26 | |
| 1 | 33 | 3 | 26.92 | 1297 | 1260 | 37.1 | 84.67 | 17 | 752582 | MH27 | |
| 1 | 33 | 3 | 27.11 | 1215 | 1179 | 37.05 | 86.37 | 8 | 713387 | MH28 | |
| 1 | 33 | 4 | 27.10 | 1221 | 1180 | 36.97 | 86.17 | 13 | 713580 | MH29 | |
| 1 | 34 | 2 | 26.94 | 1189 | 1153 | 36.76 | 91.24 | 4 | 670560 | MH30 | |
| 1 | 34 | 2 | 27.11 | 1235 | 1198 | 36.58 | 85.92 | 6 | 712737 | MH31 | |
| 1 | 33 | 4 | 26.98 | 1156 | 1116 | 41 | 99.85 | 15 | 659123 | MH32 | |
| 1 | 33 | 2 | 26.88 | 1233 | 1194 | 37.09 | 88.67 | 9 | 718721 | MH33 | |
| 1 | 34 | 2 | 26.94 | 1193 | 1152 | 36.87 | 86.66 | 9 | 670194 | MH34 | |
| 1 | 33 | 3 | 27.11 | 1217 | 1180 | 36.87 | 86.66 | 7 | 714107 | MH35 | |
| 1 | 33 | 3 | 27.11 | 1217 | 1180 | 36.9 | 85.62 | 10 | 714122 | MH36 | |
| 1 | 33 | 2 | 26.87 | 1239 | 1201 | 37.08 | 88.01 | 15 | 721499 | MH37 | |
| 1 | 33 | 2 | 27.23 | 1221 | 1185 | 36.76 | 80.94 | 11 | 714605 | MH38 | |
| 1 | 33 | 3 | 27.11 | 1216 | 1180 | 36.78 | 84.07 | 7 | 713686 | MH39 | |
| 1 | 33 | 3 | 27.11 | 1218 | 1181 | 36.96 | 86.01 | 8 | 713829 | MH40 | |
| 1 | 33 | 2 | 26.93 | 1238 | 1202 | 36.65 | 87.03 | 8 | 699120 | MH41 | |
| 1 | 33 | 2 | 27.11 | 1215 | 1179 | 36.93 | 86.73 | 7 | 713407 | MH42 | |
| 1 | 33 | 2 | 27.11 | 1219 | 1183 | 36.84 | 85.71 | 3 | 713984 | MH43 | |
| 1 | 33 | 2 | 27.16 | 1176 | 1142 | 37.19 | 89.05 | 4 | 681339 | MH44 | |
| 1 | 33 | 2 | 27.10 | 1216 | 1178 | 36.93 | 86.87 | 6 | 713131 | MH45 | |
| 1 | 33 | 2 | 27.11 | 1212 | 1173 | 36.91 | 86.82 | 12 | 714450 | MH46 | |
| 1 | 33 | 2 | 27.11 | 1216 | 1179 | 36.97 | 86.46 | 12 | 714672 | MH47 | |
| 1 | 33 | 2 | 27.11 | 1218 | 1182 | 36.94 | 86.54 | 4 | 713811 | MH48 | |
| 1 | 33 | 2 | 27.16 | 1176 | 1139 | 37.39 | 88.72 | 5 | 680977 | MH49 | |
| 1 | 33 | 2 | 27.16 | 1178 | 1140 | 37.18 | 89.05 | 4 | 680759 | MH50 | |
| 1 | 31 | 3 | 27.11 | 1217 | 1179 | 37.1 | 86.67 | 5 | 713697 | MH51 | |
| 1 | 32 | 2 | 27.16 | 1173 | 1141 | 37.19 | 89.07 | 5 | 681189 | MH52 | |
| 1 | 33 | 3 | 26.85 | 1246 | 1209 | 36.79 | 84.53 | 9 | 724514 | MH53 | |
| 1 | 33 | 2 | 26.87 | 1279 | 1243 | 36.8 | 83.47 | 16 | 720838 | MH54 | |
| 1 | 33 | 2 | 27.21 | 1225 | 1189 | 36.77 | 81.96 | 11 | 700503 | MH55 | |
| 1 | 34 | 3 | 26.93 | 1222 | 1179 | 36.69 | 87.1 | 13 | 704703 | MH56 | |
| 1 | 34 | 3 | 26.93 | 1220 | 1183 | 36.73 | 87.02 | 9 | 705100 | MH57 | |
| 1 | 34 | 3 | 26.93 | 1222 | 1185 | 36.71 | 87.22 | 11 | 705232 | MH58 | |
| 1 | 34 | 3 | 26.95 | 1251 | 1213 | 36.77 | 85.85 | 14 | 718627 | MH59 | |
| 1 | 33 | 2 | 26.85 | 1224 | 1189 | 36.84 | 83.54 | 10 | 699975 | MH60 | |
| 1 | 33 | 4 | 26.83 | 1288 | 1248 | 36.83 | 79.23 | 45 | 739614 | MH61 | |
| 1 | 33 | 3 | 26.99 | 1154 | 1119 | 40.99 | 99.88 | 7 | 659213 | MH62 | |
| 1 | 33 | 4 | 2712 | - | 1113 |  |  | 1 | 665445 | PG21 | |

**Supplementary table 3: S**ummary of substitution, insertion, and deletion numbers among *Mycoplasma hominis* clinical strains.

| Strain | Number of SNP | Number of transition | Number of transversion | Number of indels (insertion and deletion) |
| --- | --- | --- | --- | --- |
| MH1 | 91 | 42 | 48 | 1 |
| MH2 | 9764 | 7993 | 1731 | 40 |
| MH3 | 7965 | 6502 | 1431 | 32 |
| MH4 | 9684 | 7851 | 1788 | 45 |
| MH5 | 6005 | 4851 | 1124 | 32 |
| MH6 | 9666 | 7835 | 1785 | 46 |
| MH7 | 9694 | 7864 | 1786 | 44 |
| MH8 | 9845 | 7992 | 1803 | 50 |
| MH9 | 9673 | 7854 | 1777 | 42 |
| MH10 | 9481 | 7741 | 1694 | 46 |
| MH11 | 9255 | 7511 | 1710 | 34 |
| MH12 | 3571 | 2879 | 678 | 14 |
| MH13 | 9845 | 7992 | 1803 | 50 |
| MH14 | 94 | 47 | 46 | 1 |
| MH15 | 10015 | 8213 | 1760 | 42 |
| MH16 | 9811 | 7970 | 1791 | 51 |
| MH17 | 97 | 50 | 46 | 1 |
| MH18 | 3573 | 2880 | 679 | 14 |
| MH19 | 7973 | 6487 | 1449 | 37 |
| MH20 | 9821 | 7969 | 1801 | 51 |
| MH21 | 9858 | 7995 | 1812 | 51 |
| MH22 | 3571 | 2880 | 677 | 14 |
| MH23 | 7090 | 5806 | 1260 | 24 |
| MH24 | 8682 | 7130 | 1520 | 32 |
| MH25 | 100 | 48 | 51 | 1 |
| MH26 | 9927 | 8093 | 1796 | 38 |
| MH27 | 10794 | 8871 | 1896 | 28 |
| MH28 | 8632 | 7092 | 1511 | 29 |
| MH29 | 8668 | 7123 | 1515 | 30 |
| MH30 | 9511 | 7753 | 1711 | 47 |
| MH31 | 9935 | 8122 | 1813 | 38 |
| MH32 | 96 | 52 | 44 | 1 |
| MH33 | 10757 | 8867 | 1891 | 28 |
| MH34 | 9432 | 7735 | 1697 | 48 |
| MH35 | 10466 | 8625 | 1841 | 32 |
| MH36 | 10458 | 8618 | 1840 | 32 |
| MH37 | 10786 | 8885 | 1902 | 29 |
| MH38 | 10226 | 8384 | 1842 | 44 |
| MH39 | 10464 | 8625 | 1839 | 32 |
| MH40 | 10461 | 8624 | 1837 | 33 |
| MH41 | 9570 | 7829 | 1741 | 44 |
| MH42 | 10467 | 8625 | 1842 | 33 |
| MH43 | 10463 | 8620 | 1843 | 32 |
| MH44 | 8291 | 6826 | 1465 | 26 |
| MH45 | 10463 | 8624 | 1839 | 33 |
| MH46 | 10444 | 8606 | 1838 | 31 |
| MH47 | 10443 | 8607 | 1836 | 31 |
| MH48 | 10472 | 8627 | 1845 | 33 |
| MH49 | 8294 | 6829 | 1465 | 26 |
| MH50 | 8289 | 6827 | 1462 | 26 |
| MH51 | 10469 | 8624 | 1845 | 33 |
| MH52 | 8303 | 6832 | 1471 | 27 |
| MH53 | 10315 | 8502 | 1813 | 37 |
| MH54 | 6000 | 4876 | 1124 | 31 |
| MH55 | 9562 | 7822 | 1740 | 47 |
| MH56 | 10021 | 8242 | 1779 | 42 |
| MH57 | 10032 | 8253 | 1779 | 42 |
| MH58 | 10027 | 8248 | 1779 | 42 |
| MH59 | 10028 | 8240 | 1788 | 39 |
| MH60 | 9986 | 8220 | 1767 | 41 |
| MH61 | 9736 | 8032 | 1708 | 39 |
| MH62 | 91 | 47 | 44 | 1 |

**Supplementary table 4**: Temporary biosample-accession number of the 62 *Mycoplasma hominis* strains

| *Mycoplasma hominis* Strains | Temporary biosample_accession number |
| --- | --- |
| MH1 | SAMN47604343 |
| MH2 | SAMN47604344 |
| MH3 | SAMN47604345 |
| MH4 | SAMN47604346 |
| MH5 | SAMN47604347 |
| MH6 | SAMN47604348 |
| MH7 | SAMN47604349 |
| MH8 | SAMN47604350 |
| MH9 | SAMN4760451 |
| MH10 | SAMN47604352 |
| MH11 | SAMN47604353 |
| MH12 | SAMN47604354 |
| MH13 | SAMN47604355 |
| MH14 | SAMN47604356 |
| MH15 | SAMN47604357 |
| MH16 | SAMN47604358 |
| MH17 | SAMN47604359 |
| MH18 | SAMN47604360 |
| MH19 | SAMN47604361 |
| MH20 | SAMN47604362 |
| MH21 | SAMN47604363 |
| MH22 | SAMN47604364 |
| MH23 | SAMN47604365 |
| MH24 | SAMN47604366 |
| MH25 | SAMN47604367 |
| MH26 | SAMN47604368 |
| MH27 | SAMN47604369 |
| MH28 | SAMN47604370 |
| MH29 | SAMN47604371 |
| MH30 | SAMN47604372 |
| MH31 | SAMN47604373 |
| MH32 | SAMN47604374 |
| MH33 | SAMN47604375 |
| MH34 | SAMN47604376 |
| MH35 | SAMN47604377 |
| MH36 | SAMN47604378 |
| MH37 | SAMN47604379 |
| MH38 | SAMN47604380 |
| MH39 | SAMN47604381 |
| MH40 | SAMN47604382 |
| MH41 | SAMN47604383 |
| MH42 | SAMN47604384 |
| MH43 | SAMN47604385 |
| MH44 | SAMN47604386 |
| MH45 | SAMN47604387 |
| MH46 | SAMN47604388 |
| MH47 | SAMN47604389 |
| MH48 | SAMN47604390 |
| MH49 | SAMN47604391 |
| MH50 | SAMN47604392 |
| MH51 | SAMN47604393 |
| MH52 | SAMN47604394 |
| MH53 | SAMN47604395 |
| MH54 | SAMN47604396 |
| MH55 | SAMN47604397 |
| MH56 | SAMN476043198 |
| MH57 | SAMN47604399 |
| MH58 | SAMN47604300 |
| MH59 | SAMN47604301 |
| MH60, | SAMN47604302 |
| MH61 | SAMN47604303 |
| MH62 | SAMN47604404 |

**Source:** GenBank submission ID: SUB15204443, bioproject number: PRJNA1242796

**Supplementary figures**

**
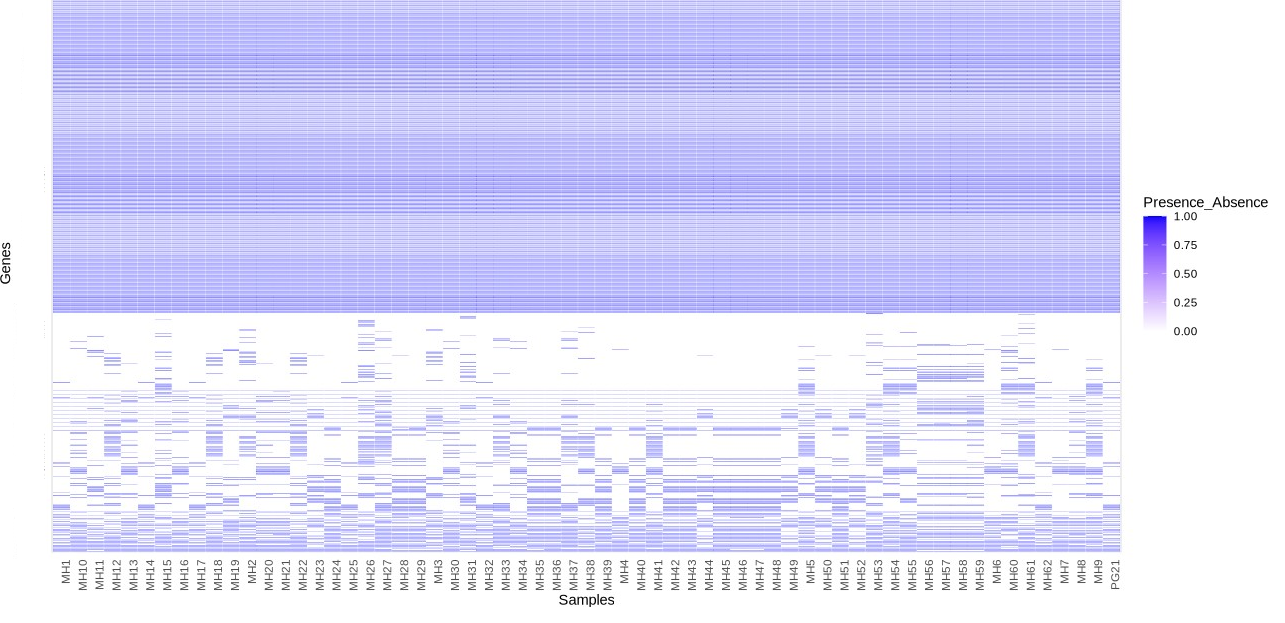
**

**Supplementary figure 1.** Pangenomic matrix of pangenome genes. Present genes are in blue color and absent genes are in white color.

**(A)**


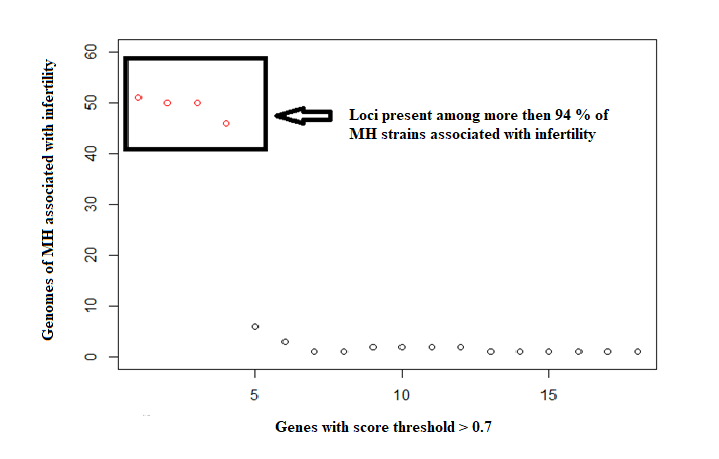


**Supplementary figure 2 (A).** R package plot presentation of genes associated with infertility pathotype among *Mycoplasma hominis* clinical strains. Red and Black dots correspond to genes present in more than 94 % of strains and in less than 20 % of *M. hominis* strains, respectively

**(B)**

**
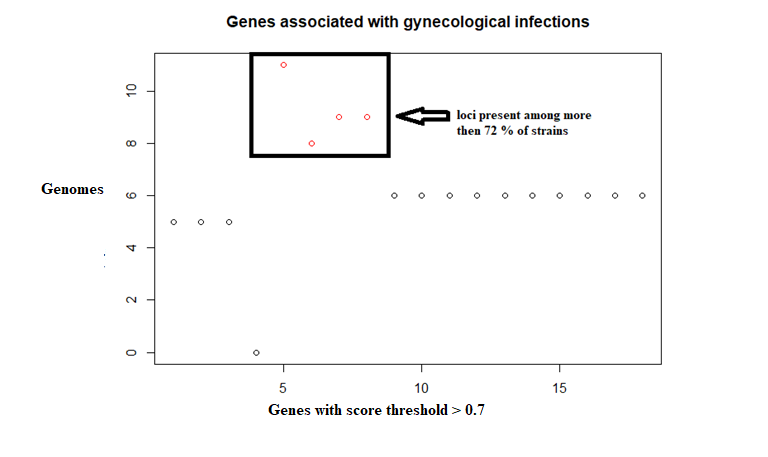
**

**Supplementary figure 2 (B):** R package Plot representing genes associated with gynecological infections pathotype. Red dots represent Asparagine tRNA ligase, restriction endonuclease subunit S, Eco47II family restriction endonuclease, and transcription regulator XRE. While black dots represent genes that are present in less than 60 % of *M. hominis* strains assigned to gynecological infections pathotype.
